# Flexible ureterorenoscopy and laser lithotripsy with regional anesthesia vs general anesthesia: A prospective randomized study

**DOI:** 10.1590/S1677-5538.IBJU.2019.0770

**Published:** 2020-09-02

**Authors:** Murat Sahan, Omer Sarilar, Mehmet Fatih Akbulut, Eren Demir, Metin Savun, Oznur Sen, Faruk Ozgor

**Affiliations:** 1 Department of Urology Haseki Training and Research Hospital Istanbul Turkey Department of Urology, Haseki Training and Research Hospital, Istanbul, Turkey;; 2 Department of Anesthesiology and Reanimation Haseki Training and Research Hospital Istanbul Turkey Department of Anesthesiology and Reanimation, Haseki Training and Research Hospital, Istanbul, Turkey

**Keywords:** Lithotripsy, Laser, Anesthesia, General, Urolithiasis

## Abstract

**Purpose:**

To compare the effect of general anesthesia (GA) and regional anesthesia (RA) on f-URS outcomes and surgeon comfort.

**Material and Methods:**

The study was conducted between June 2017 to January 2018 and data collection was applied in a prospective, randomized fashion. 120 patients participated in the study and were divided into RA group (n=56) and GA group (n=64). Demographic, operative and post-operative parameters of patients were analysed. The end point of this study was the effect of two anesthesia regimens on the comfort of the surgeon, and the comparability of feasibility and safety against perioperative complications.

**Results:**

The study including 120 randomized patients, 14 patients were excluded from the study and completed with 106 patients (45 in RA group and 61 in GA group). No difference was detected between the two groups in terms of preoperative data. During the monitorization of operative vital signs, 3 patients in RA group experienced bradycardia, and this finding was significant when compared with GA group (p=0.041). Additionally, 2 patients in RA group experienced mucosal tears and 1 patient experienced hemorrhage during the operation, but no complications were observed in the GA group (p=0.041). Postoperative surgeon comfort evaluation revealed statistically significant results in favor of GA group (p=0.001).

**Conclusions:**

Both GA and RA are equally effective and safe anesthesia methods for f-URS procedures. However, RA group showed significantly increased likelihood of bradycardia and mucosal injury during surgery, and significantly decreased surgeon comfort during surgery.

## INTRODUCTION

Urolithiasis is a widespread disorder all around the worldwide and almost 10% of the population faces urolithiasis related health problems during their life-span ( 1 ). Flexible ureterorenoscopy (f-URS) is the state of art, and accepted as an important approach in the management of renal stones because of its reasonable success and lower complication rates ( 2 , 3 ). There has been an extensive examination of factors affecting f-URS success, such as stone volume, stone location, stone number and surgeon experience, however; effect of anesthesia type has not been evaluated sufficiently ( 4 , 5 ).

Anesthesiologists and urologists usually prefer to perform renal stone surgeries (RSS) under general anesthesia, however, previous reports demonstrated RSS could be performed both under general anesthesia (GA) and with regional anesthesia (RA) ( 6 , 7 ). Control of anesthesia duration, effective control of patient’s respiratory movements and high patient’s compliance are the advantages of the GA during RRS ( 8 , 9 ). Aspiration of gastric contents, adverse drug events and cardiopulmonary complications are more common in patients undergoing GA ( 10 ). On the other hand, risk of venous embolism and bleeding are lower in patients undergoing RA ( 11 ).

Although many studies have investigated the possible predictive factors which may have an effect on f-URS outcomes, the role of anesthesia type has not been evaluated sufficiently. The present study was the first to compare the effect of general anesthesia (GA) and regional anesthesia (RA) on f-URS outcomes and surgeon comfort.

## MATERIALS AND METHODS

The study was conducted between June 2017 to January 2018 and data collection was applied in a prospective, randomized fashion. Ethical committee approval and patient’s written informed consents were obtained. Patients with renal stone between 18 to 60 years of age, with American Society of Anesthesiologists (ASA) physical status score of 1-2 were included in the study. Exclusion criteria were history of cardiac, respiratory, neuromuscular disease, pregnancy, congenital renal anomalies, contraindications of regional or general anesthesia such as skin infection of back, vertebral deformity, and neuropathy. After inclusion and exclusion criteria were applied, 120 patients participated in the study. Before the induction of anesthesia, a coin was flipped accompanied by surgical nurse and heads were included into RA group, tails were included into GA group. Thus, these patients were divided into RA group (n=56) and GA group (n=64) by a simple random sampling method, tossing a coin. The end point of the study was planned for 8 months.

### Anesthesia Technique

All patients received I.V. premedication with 0.05mg/kg midazolam and a 50mL normal saline solution in the preoperative care unit. After patients were taken to the operating room, initial blood pressure and heart rate measurements were recorded as baseline. After the patients were seated at the operation table, the skin surface of the back was cleaned and sterilized with 10% povidone iodine. A combined spinal epidural set (18G epidural and 27G intrathecal needles) and 0.5% heavy bupivacaine were used for RA. Then, 3mL of 2% lidocaine was injected into the skin and the subcutaneous tissue. The loss-of-resistance method was used to find the epidural space at L 2-3 or L 3-4 vertebrae, and 15mg of 0.5% bupivacaine heavy was given to intrathecal space. After the spinal block, an epidural catheter was inserted 5cm inside and fixed to the skin surface. Motor block was assessed according to the modified Bromage scale; 0, no motor block; 1, hip blocked; 2, hip and knee blocked; 3, hip, knee and ankle blocked. If an adequate level of sensation was achieved, the operation was begun; if not, conversion to GA was applied and the patient was excluded from the study. If there were any signs of regression of block, or if the patient felt pain, 5mL of 0.5% bupivacaine was administered to the epidural catheter. All drugs and doses administered during the operation were recorded. After the operation, patients were transferred to the postoperative care unit (PACU).

One µg/kg fentany l, 2mg/kg propofol, 0.6mg/kg rocuronium were used for induction of the GA. Oro-tracheal intubation was performed after adequate muscle relaxation was achieved. Anesthesia administration was achieved with 60% oxygen, 2 l/min flow rate, 0.8% to 3% sevoflurane. At the end of the operation, 1gr paracetamol and 1mg/kg tramadol I.V. were administered. If muscle relaxation was detected during the operation, rocuronium 0.15mg/kg I.V. were applied. The neuromuscular block with atropine (0.01mg/kg) and neostigmine (0.02mg/kg) was reversed after the operation was terminated. The patients were extubated when adequate spontaneous ventilation was detected, and then transferred to the PACU. Patients with a Modified Aldrette score of 9 were transferred to the in-patient clinic from the PACU. The length of stay in PACU was recorded.

**Figure 1:**
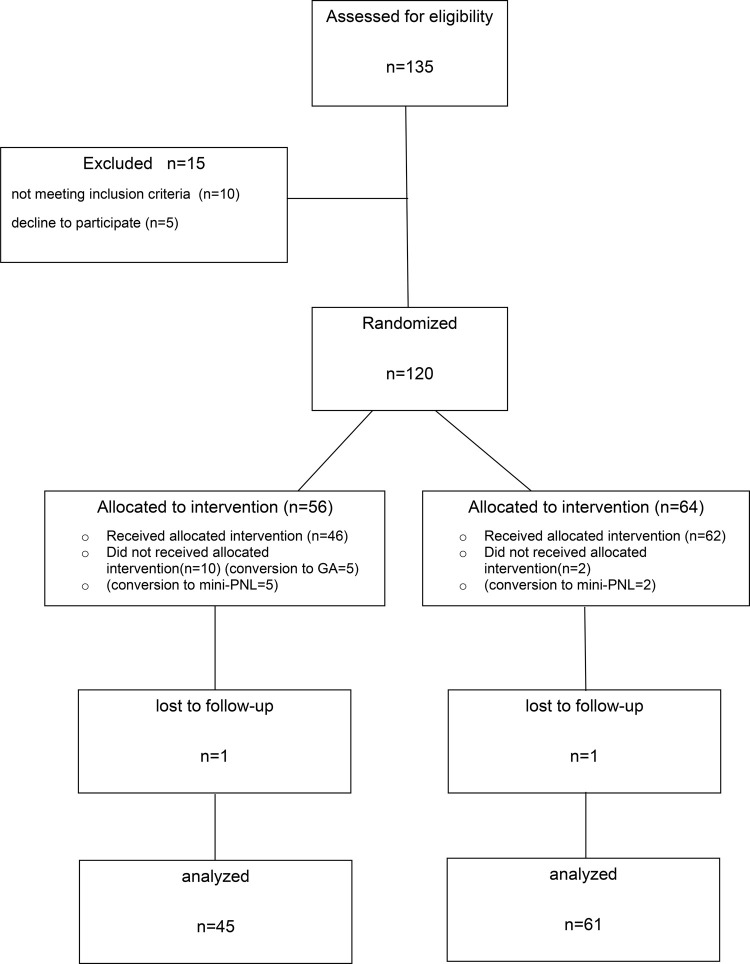
Consort diagram showing study design.

All patients were visited before the day of the operation day, informed about the study and given an explanation of the Visual Analog Scale (VAS) score, where ‘0’ score corresponds to no pain, and ‘10’ to maximum or worst pain. The postoperative pain was assessed by using dynamic and static VAS. VAS scores were recorded at 1, 3, 6, 12 and 24 hours after surgery. In the first 24 hours, if the VAS score was more than 6 points, the patient was given a maximum of 300mg/day tramadol from 1mg/kg and the total amount of tramadol given was recorded. Patients who experienced pain but had a VAS score below 6 points were given diclofenac sodium 75mg I.M. to a maximum dose of 150mg. When the pain persisted, tramadol was injected and the amount was recorded. At 24 hours postoperatively, patient satisfaction was scored from 1 to 5 (1-very bad, 2-bad, 3-moderate, 4-good, 5-very good).

### Operation Technique

For patients with/without preoperative stenting, a 9.5 French (Fr) semi-rigid URS was performed for optical dilatation and to visualize the entire ureter. Then, a guidewire was inserted into ureter and 11/13Fr ureteral access sheath was placed in all cases. The intrarenal collecting system was visualized with 7.5/8.5Fr flexible ureteroscope (Flex X2, Storz, Tuttlinger, Germany), and holmium laser with a 272µm fiber was used for laser lithotripsy. Nitinol baskets were used at the end of the lithotripsy to remove fragments from the collecting system, at surgeon’s discretion. Ureteral double J stent was inserted to every patient and removed 2 weeks after the operation.

Demographic data of patients and stone characteristics, operation time, fluoroscopy time, length of hospital stay and stone-free rates (SFR) were recorded. Perioperative number of hypotension, hypertension, tachycardia and bradycardia were recorded. Anesthesia-related side-effects in patients (nausea, vomiting, pruritus and respiratory depression) were noted. On first postoperative day, kidney-ureter-bladder (KUB) radiography was obtained to evaluate the localization of double j stent and residual stones. SFR was re-evaluated with non-contrast computed tomography (NCCT) after the first postoperative month. Success was considered as residues of <2mm or absence of any stone fragments. Moreover, the parameters affecting the comfort of the surgeon such as ergonomics, comfort of being sure about the safety of the patient, difficulty of laser focusing during surgery were assessed by the surgeon, scoring between 1 (very poor) and 10 (very good). The end point of this study was the effect of two anesthesia regimens on the comfort of the surgeon, and the comparability of feasibility and safety against perioperative complications.

### Statistical Analysis

Patient’s demographic characteristics (age, sex, etc.), stone dimensions, operation data, complications, and postoperative success status were evaluated separately for both groups (GA and RA). The necessary sample size was calculated to be 78 patients (39 per group), the power of study was 80, alpha value was 0.05. This data was analyzed with Statistical Package for the Social Sciences (SPSS) 20 program. For the analysis of quantitative data, the normal distribution suitability was examined by the Kolmogrov-Simirnov test; parametric methods were used in the analysis of normal distribution variables, and non-parametric methods were used in the analysis of variables not showing normal distribution. Independent t test was used to compare independent groups, Pearson correlation test was used to examine the relationship between variables, and Pearson chi-square, chi-square and Fisher exact tests were used to compare categorical data. Quantitative data are expressed as mean±std values on tables. Categorical data are expressed as n (frequency) and percentages (%). Data were analyzed at 95% confidence level, and was considered significant when the p value was less than 0.05.

## RESULTS

The study included 120 randomized patients, 14 patients were excluded from the study and completed with 106 patients (45 in RA group and 61 in GA group). Five patients were excluded from the study because of the failure of RA and conversion to GA was required. One patient in RA and GA group was lost to follow-up and was excluded. Three patients in RA group and two patients in GA group were converted to mini-percutaneous nephrolithotomy (PNL) procedure because of the failure of ureteral access sheath placement due to ureteral stricture. Two patients in RA group were excluded from the study because of acute infundibulum-pelvic angle and narrow infundibulum causing the inability to reach the stone who were converted to mini-PNL procedure.

No difference was detected between the two groups in terms of age, sex, ASA score, stone size and localization, operation side or history of previous RSS. Patient’s preoperative data are listed in Table-1 .

**Table 1:** Comparison of preoperative demographic data of patients.

	Groups		

	General Anesthesia	Regional Anesthesia	P value
**Number**	61	45	
Gender (Male/Female)	35/26	26/19	0.967
Age* (years)	46±16.3	44.1±12.6	0.520
ASA Score*	1.3±0.5	1.4±0.5	0.449
Hydronephrosis Grade*	1.2±0.8	1.2±0.6	0.832
Stone size* (mm)	17.2±7.7	15.7±7.3	0.326
**Stone Location**			0.656
Upper Pole	6 (9.8%)	1 (2.2%)	
Middle Pole	3 (4.9%)	10 (22.2%)	
Lower Pole	9 (14.8%)	9 (20%)	
Renal Pelvis	26 (42.6%)	12 (26.6 %)	
Multiple	17 (27.9%)	13 (28.9%)	
Operation side (Right/Left)	30/31	25/20	0.150
**Previous Stone Treatment**			0.781
SWL	10	9 (20%)	
PNL	6 (9.8%)	5 (11.1%)	
URS/f-URS	5 (8.2%)	7 (15.6%)	
Open Surgery	4 (6.6%)	0	
Multiple Surgery	13 (21.3%)	6 (13.3%)	

* = Mean; **ASA score** = American Society of Anesthesiologists Score; **SWL** = Shock Wave Lithotripsy; **PNL** = Percutaneous Nephrolithotomy; **URS** = Ureterorenoscopy; **f-URS** = Flexible Ureterorenoscopy

The RA group mean operation time was longer but the difference did not reach statistical significance (59.2±19.6 min in Group I, 53.8±21.7 min in Group II, p=0.186). The mean fluoroscopy time was 0.3±0.6 min in GA group and 0.4±0.9 min in RA group (p=0.229). The mean duration of hospital stay was similar between groups. In terms of stone clearance at the 1-month visit with NCCT; complete clearance was achieved in 86 of 106 patients, and no significant difference was detected between groups (77% in GA group, 86.7% in RA group). During the monitorization of operative vital signs, 3 patients in RA group experienced bradycardia, and this finding was significant when compared with GA group (p=0.041). Additionally, 2 patients in RA group experienced mucosal tears and 1 patient experienced insignificant hemorrhage but it however made vision, and hence the procedure itself, was considered difficult during the operation. Nevertheless, no complications were observed in the GA group (p=0.041) ( Table-2 ).

**Table 2:** Comparison of perioperative parameters and outcomes.

	Groups		

	General Anesthesia	Regional Anesthesia	P value
**Number**	61	45	
Operation time (min.)*	53.8±21.7	59.2±19.6	0.186
Fluoroscopy time (min.)*	0.3±0.6	0.4±0.9	0.229
Hospitalization time (hours)*	36.6±2.6	36.8±5.4	0.791
Perioperative tachycardia	0	0	-
Perioperative bradycardia	0	3 (6.7%)	0.041
Perioperative hypertension	0	0	-
Perioperative hypotension	0	0	-
**Perioperative complications**			0.041
Hemorrhage	0	1 (2.2%)	
Mucosal tear	0	2 (4.4%)	
Perforation	0	0	-
Stone free status	47 (77%)	39 (86.7%)	0.215

* = Mean

Postoperative static VAS scores in GA and RA groups were 2.3±1.6 vs. 2.7±1.7 at 3rd hour, 1.6±1.8 vs. 2.2±2.1 at 6th hour, 0.9±0.9 vs. 1.3±1.0 at 12th hour, 0.7±0.8 vs. 0.8±0.9 at 24th hour, respectively. No significant difference was detected among groups in terms of postoperative static VAS scores. Additionally, no difference was detected among groups in terms of postoperative need for analgesia. During postoperative follow-up, nausea and vomiting was observed in 1 GA group patient, whereas there was no such complication in RA group. No itching or respiratory depression was detected in any patient. In RA group, 2 patients required antibiotic treatment for urinary tract infection, which was accompanied by fever. Patient satisfaction rates elicited at postoperative 24th hour, revealed similar results between groups. Postoperative surgeon comfort evaluation revealed statistically significant results in favor of GA group (p=0.001). Duration of stay in PACU were similar between groups ( Table-3 ).

**Table 3:** Comparison of postoperative parameters and outcomes.

	General Anesthesia	Regional Anesthesia	P value
Static VAS Score*			
Post op 1. hour	2.5±1.4	2.9±1.9	0.171
Post op 3. hours	2.3±1.6	2.7±1.7	0.261
Post op 6. hours	1.6±1.8	2.2±2.1	0.147
Post op 12. hours	0.9±0.9	1.3±1	0.125
Post op 24. hours	0.7±0.8	0.8±0.9	0.504
Dynamic VAS Score*			
Post op 1. hour	2.5±1.4	3±2.9	0.153
Post op 3. hours	2.3±1.6	2.7±1.7	0.290
Post op 6. hours	1.7±1.8	2.2±2.3	0.138
Post op 12. hours	1±0.9	1.3±1.1	0.107
Post op 24. hours	0.7±0.8	0.8±0.9	0.572
Tramadole requirement first 24 hours	16 (26.2%)	14 (31.1%)	0.581
NSAID requirement first 24 hours	32 (52.5%)	26 (57.8%)	0.166
Nausea-vomiting first 24 hours	1 (1.6%)	0	0.393
Itching first 24 hours	0	0	-
Respiratory depression first 24 hours	0	0	-
Patient satisfaction after 24 hours*	4.4±0.6	4.3±0.6	0.311
Duration in PACU (min)*	4.6±1	4.5±0.5	0.516
Fever requiring antibiotic therapy in the first 24 hours	0	2 (4.4%)	0.183
Surgeon comfort*	7.6±1.1	6.3±1.5	0.001

* = Mean; **VAS** = Visual Analogue Scale; **NSAID** = Nonsteroidal Anti-inflammatory Drug **; PACU** = Post-Anesthesia Care Unit

## DISCUSSION

General anesthesia is the preferred anesthesia type for f-URS in majority of studies in the literature ( 5 , 6 , 8 , 12 ). RA has been shown to be safe and effective in the treatment of renal stones in PNL procedure. However, few studies have evaluated the efficacy of RA during f-URS. Both anesthesia types have advantages and disadvantages in terms of surgery success, complication rates and patient and surgeon comfort. Effective control of respiratory movements can be stated as an advantage of GA; however, patients who received RA experienced fewer hemorrhagic complications and thromboembolic events, shorter operation duration and less postoperative pain ( 11 ). Thus, there is no consensus on the recommended type of anesthesia for f-URS procedure.

Pain after RSS deteriorates patient’s quality of life and also prolongs the hospital stay, and increases the amount of analgesics used and overall cost. Tangpaitoon et al. ( 10 ) evaluated the effect of anesthesia type during RSS on patient’s postoperative pain, noting that those who received RA experienced less pain at 1st and 4th hour when compared with those who received GA (p <0.001 and 0.025, respectively). Singh et al. ( 13 ) stated that on the first postoperative day, patients receiving RA during PNL experienced less pain than those receiving GA. However, they found no significant difference in terms of pain on the second postoperative day. Kim et al. ( 14 ) and Cakici et al. ( 12 ) found no difference in pain levels between the two different anesthesia types during RSS. In our study, we found no significant difference between GA and RA in terms of postoperative pain among patients undergoing f-URS procedure. According to our results, RA appears as an acceptable alternative to GA, with similar levels of postoperative pain.

The main aim of the treatment of nephrolithiasis patients is to achieve complete stone clearance with minimal morbidity, by using minimally invasive treatment modalities. In our study, complete stone clearance was achieved in 77% of patients in GA group and 86.7% of patients in RA group. No significant difference was detected among groups in terms of stone clearance (p=0.215). Zeng et al. ( 15 ) performed f-URS in a total of 65 patients under GA (n=34) and RA (n=31). Similar to our results, their stone clearance rates were 70.6% in GA and 67.7% in RA group, not significantly different. In two studies, conducted by Kim et al. and Kuzgunbay et al. ( 14 , 16 ) respectively, no significant difference in terms of stone clearance was detected between patients who underwent PNL under GA or RA. In accordance with the literature, our study also revealed no effect of anesthesia type on stone clearance rates.

In the current study, in RA group, mucosal tear and hemorrhage occurred in 4.4% and 2.2% of patients respectively. In terms of perioperative complications, a significant difference was observed between our groups (p=0.041). We emphasize that this difference was due to inadequate stabilization of respiratory muscles during RA, and thereby, difficulty of laser focusing during stone fragmentation due to increased mobility of the kidney during surgery. The incidence of bradycardia was also significantly higher in RA group. In contrast, Zeng et al. ( 15 ) found no significant difference between RA and GA groups in terms of operative complications, nor any mucosal injury due to increased mobility of the kidney. Additionally, they found increased likelihood of bradycardia during RA, but this difference was not statistically significant. Karacalar et al. ( 17 ) found similar results with our study, in terms of the extent of postoperative vomiting and itching in both groups. However, they found increased patient satisfaction in RA group, while in our study, patient satisfaction rates were similar between groups.

Even though RA has advantages such as shorter hospital stay and fewer thromboembolic complications, it also has certain disadvantages such as spontaneous breathing and deep inspirations during surgery with effects on the surgical field, inability to suppress coughing and sneezing reflexes, or failure to prevent patient movement during surgery due to ineffective analgesia, all of which may affect surgeon’s comfort negatively during surgery. In GA, the elimination of these disadvantages can positively effect parameters such as ergonomics and laser focusing, thus improving the comfort of the surgeon. The present study was the first to study evaluating the surgeon’s comfort, which was found to be significantly better in GA group.

This study was the first to evaluate anesthesia type in f-URS cases in terms of success, complications and surgeon comfort; however, it has some limitations. First of all, patient volume was relatively small. Additionally, the effect of anesthesia type on operation duration was evaluated, but not its effect on the duration of stone fragmentation. The study assessed postoperative pain in the first 24 hours, but not the long term effect of anesthesia type on experienced pain. Finally, the cost-effectiveness of these two anesthesia types were not evaluated and should be the focus of further studies.

In conclusion, both GA and RA are equally effective and safe anesthesia methods for f-URS procedures. However, RA group showed significantly increased likelihood of bradycardia and mucosal injury during surgery, and significantly decreased surgeon comfort during surgery. Further prospective randomized studies with larger patient volume will bring more detailed insights.

## ABBREVIATIONS

f-URS: Flexible Ureterorenoscopy
RSS: Renal Stone Surgeries
GA: General Anesthesia
RA: Regional Anesthesia
ASA score: American Society of Anesthesiologists Score
PACU: Postoperative Care Unit
VAS: Visual Analog Scale
PNL: Percutaneous Nephrolithotomy
SWL: Shock Wave Lithotripsy
URS: Ureterorenoscopy
